# The Need for Alcohol Abuse-Related Education in Nursing Curricula

**Published:** 1994

**Authors:** Madeline A. Naegle

**Affiliations:** Madeline A. Naegle, R.N., Ph.D., F.A.A.N., is an associate professor in the Division of Nursing, School of Education and the project director of Substance Abuse Education in Nursing (SAEN), New York University, New York, New York

## Abstract

Because nurses play a prominent role in primary care, they regularly encounter and attend people with alcohol abuse and dependence problems. For that reason, it is vital that education about alcohol abuse and related problems, which is currently offered in a sporadic manner, becomes an integral element of basic nursing curricula.

Primary care includes providing patients with guidelines and information for maintaining good health as well as providing the screening, diagnosis, intervention, and treatment of basic health problems, including alcohol abuse and dependence. Nurses play a crucial role in the primary care setting. They assess client needs; formulate and deliver care to individuals and families; and often are responsible for detecting, addressing, and referring patients who exhibit alcohol, tobacco, and other drug-related^1^ problems.

Yet basic knowledge about alcohol abuse and its related problems has not been included in general nursing education in any consistent manner. Indeed, significant gaps exist in the content of nursing curricula aimed at educating nurses about substance abuse.

This article briefly reviews the role of nurses in primary care; examines the history of alcohol abuse nursing curricula; describes some model curricula programs; considers the need for clinical placement, preceptorships, and faculty development; and discusses efforts to standardize alcohol abuse curricula.

## Current Nursing Education

Basic nursing education now takes place in two major degree programs. The 2-year associate degree program teaches nurses to provide direct patient care from a base of scientific knowledge and liberal arts. The 4-year baccalaureate degree program prepares nurses to implement prevention, treatment and long-term strategies, such as conducting interviews to obtain patients’ histories and taking physical assessments. Nurses with baccalaureate degrees function with more autonomy and have a greater depth of knowledge than do nurses with associate degrees.

A master’s degree in a specific content area allows nurses to specialize. Primary care nurse practitioners, nurse midwives, and addictions specialist nurses have master’s degrees and are prepared and proficient at nursing diagnosis and health care management within their chosen areas.

## Roles of Nurses in Primary Care

Primary care activities related to alcohol abuse center on the early prevention and intervention of problems and on the identification of problems and referral to specialty care.

Such an emphasis on prevention provides opportunities for generalist^2^ nurses to assume more formal roles in providing primary care. Having a basic nursing education prepares nurses to identify alcohol-related problems in patients and to refer them to treatment specialists. For nurse practitioners, the role extends beyond primary care to include secondary care of patients with alcohol abuse and dependence (primary care referring to prevention and health maintenance and secondary care to diagnosis and care of acute illnesses).

A primary care nurse addressing alcohol-related problems must have received adequate education and training to obtain routine alcohol and other drug histories; implement primary prevention strategies, including anticipatory guidance and alcohol abuse education; assess a possible problem with alcohol; formulate a diagnosis of abuse from an analysis of patient assessments and data collection (e.g., laboratory results); conduct appropriate nursing interventions (e.g., patient education and nutrition counseling); identify acute alcohol-related illness and refer patients to physicians or addictions nursing specialists; and continue care in the forms of followup, monitoring, health maintenance, or health care support during recovery.

## The Evolution of Nursing Education

Information about alcohol abuse and its related problems has not been included consistently in nursing curricula. Long-term effects of alcoholism and appropriate nursing care for this condition often were included in professional nursing curricula developed in the 1940’s. Yet it was not until 1977 that a textbook—*Alcoholism: Development, Consequences and Interventions*, by Estes and Heinemann—consolidated existing scientific findings and clinical observations to provide specific implications about alcohol abuse to the nursing community.

After the publication of that text, authors of other nursing textbooks in psychiatric, mental health, and community health nursing began to present chapters on alcohol abuse and dependence, including its health implications and related medical problems. However, the first textbook devoted to the abuse of substances, including alcohol—*Substance Abuse*, by Bennett and Woolf—was not published until 1991.

Even with the inclusion of alcohol abuse content in textbooks, a 1987 survey by Hoffman and Heinemann indicated that little expansion of education about alcohol abuse in nursing curricula had occurred. Nursing schools averaged only 1 to 5 hours on alcohol abuse education, a disproportionately small amount given its far-reaching health implications. Content related to alcohol abuse and dependence was presented primarily in courses on psychiatric or medical nursing, as associated with long-term effects and medical complications.

## Early Efforts in Curriculum Development

In the late 1970’s, various efforts recognized deficits in nursing education for alcohol-related problems. In conjunction with the National Center for Alcohol Education, the National Institute on Alcohol Abuse and Alcoholism (NIAAA) published *The Community Health Nurse and Alcohol Related Problems: A Book of Readings* (1978). This was followed by the inclusion of alcohol-abuse nursing curricula in the Health Professions Education, Curriculum Resources Series published by NIAAA and the National Institute on Drug Abuse (NIDA).

The publication *Alcohol Abuse Curriculum Guide for Nurse Practitioner Faculty* (Hasselblad 1984) describes nursing practice based on knowledge of alcohol abuse and dependence in a manner specific to the nurse practitioner’s role. The book had its origins in the Conference of Nurse Educators on Alcohol and Drug Abuse held in Colorado Springs in 1982.

## Developing Curriculum Models

In 1988, a federally funded project promised to expand and standardize alcohol and other drug nursing curricula. NIAAA, NIDA, and what was then the Office of Substance Abuse Prevention (now the Center for Substance Abuse Prevention [CSAP]), jointly funded three programs to develop and evaluate model alcohol and other substance abuse curricula for undergraduate and master’s level nursing programs.

The three schools to receive funding were the Division of Nursing, School of Education at New York University; the College of Nursing at Ohio State University; and the School of Nursing at the University of Connecticut. Project guidelines allowed for some variability to reflect the schools’ unique resources or special faculty expertise, but each project had the following minimal requirements:

- To convene a working committee of alcohol abuse and dependence experts
- To develop a model curriculum on alcohol abuse in module format with stated objectives, content outlines, bibliographies, and learning resources
- To formulate learning objectives for baccalaureate and master’s degrees
- To develop module content based on scientific knowledge and research findings
- To initiate curricular changes at departmental and school levels
- To pilot test, review, and evaluate modules as components of the curriculum.

Each curriculum bases its definition of nursing on the interrelationships of patient, nurse, and environment (also see the box on p. 155). In addition, all address the needs of individuals, families, and groups in relation to alcohol abuse at each stage of human growth and development, from fetal life to old age.

**Summary of Alcohol and Other Drug Abuse Curricula for Nurses**

**Table:** 

		Module/Curriculum	Project/Source

Registered Nurses (e.g., occupational health, ambulatory care, general hospital)	Screening	Risks of alcohol and other drug abuse	NEADA^1^
		Chemical dependence assessment	Ohio State
		Assessment of the adult client	SAEN^2^
		Psychosocial effects of chemical abuse	Ohio State
		Pharmacology of major drugs of abuse	SAEN
		Dimensions of alcohol and other drug abuse	NEADA

	Interventions	Dysfunction patterns in families	SAEN
		Addictions: Diagnosis, withdrawal, intoxication	SAEN
		Continuum of treatment: Management of alcohol and other drug abuse	NEADA
		Interventions with families	Ohio State

	Prevention	Introduction to ATOD:^3^ A focus on prevention	Ohio State
		Attitudes toward alcohol and drug abuse	SAEN
		Health implications of drug and alcohol use	SAEN
		Prevention of ATOD problems in the school-age child	SAEN
		Health promotion and risk reduction	Ohio State
		Prevention	CTS Training^4^

Nurse Practitioners	Health maintenance/Continuing care	Care for the caregivers	NEADA
		Understanding, preventing, and treating ATOD	NEADA
		Central nervous system effects of psychoactive drugs	Ohio State
		Perspectives on drug and alcohol problems	SAEN
		
Midwives		Fetal effects of maternal drug and alcohol use	SAEN

1 NEADA—Nursing Education in Alcohol and Drug Abuse.

2 SAEN—Substance Abuse Education in Nursing.

3 ATOD—Alcohol, Tobacco, and Other Drugs.

4 CTS—Center for Substance Abuse Prevention Training System.

The individual curriculum contains information considered essential to the practice of the registered professional nurse who is educated at the baccalaureate level, as well as material considered central to the practice of the addictions nursing specialist. Although each curriculum contains more information than can be incorporated into an associate degree program, they all can be adapted to meet the needs of students at levels of practice considered more technical than professional. Furthermore, most modules can be taught separately as continuing education for graduate nurses.

### Specifics From Three Schools

Ohio State’s College of Nursing curriculum modules are the most detailed of the three schools, especially in the way that the assessment and organization of content are related to learner needs, educational setting, and presentation planning. The modules cover both beginning and advanced levels of nursing preparation. The faculty at Ohio State also developed an extensive glossary of terms for use in the program.

The University of Connecticut’s Nursing Education in Alcohol and Drug Abuse project (Project NEADA) produced a two-volume curriculum containing eight modules: one for faculty development, three for undergraduates, one for postbaccalaureate students, and three for graduate students. In addition, the faculty work group leading curriculum development produced videocassette tapes to teach techniques for identifying drug and alcohol abusers. These “trigger tapes” provide scenarios and key concepts involved in interviewing patients and obtaining alcohol and other drug histories.

New York University’s Substance Abuse Education in Nursing program (Project SAEN) developed 23 modules: 15 at the beginning and advanced undergraduate levels and 8 at the graduate level. One-third of the modules are suitable for teaching students from other health disciplines and also can be adapted for continuing education. This curriculum includes modules that address the needs of special populations; the use of teams composed of health professionals from medicine and nursing, social workers, and others in addiction treatment; and the relevant research issues and methodologic considerations for research in alcohol abuse.

## Beyond Didactic Content

Clinical supervision, preceptorships, and internships are cornerstones for skill building in many health professions. A primary care provider who has skills in assessing alcohol abuse problems is of particular importance as a role model for students. Because patients usually see health care providers for reasons other than their addictions, the skills learned from a good role model are essential. Only sensitive interviewing and knowledgeable interpretation of screening tools, assessments, and laboratory data will uncover alcohol or other drugs as the cause of the patient’s primary complaint.

Model curricula are most effective when they are used in the following ways:

- Instructors integrate information on alcohol abuse into other courses, where and when it is germane to course content.
- Faculty with clinical skills in detecting and treating alcohol and other drug abuse and addiction act as role models in care settings.
- Students are placed in clinical settings, such as hospitals or health care facilities, where patients report use and manifest signs and symptoms of abuse and early, middle, and late stages of alcoholism and other addictions.
- The planning of nursing care includes interventions appropriate to the patient’s needs, ranging from health education and prevention for patients at high risk to identification of a problem followed by action, which may include referral to a specialist or counseling by the nurse.

## Faculty Development Programs

The goal of faculty development programs funded by NIAAA, NIDA, and CSAP is to train groups of medical, nursing, social work, and psychology faculty so that they may teach about alcohol and other drug abuse. These programs target faculty members whose specialty areas are other than alcohol abuse, such as family practice, adult health nursing, or psychiatry. Participants learn content on which to develop courses; integrate alcohol and other drug knowledge into standing curricula; provide consultation within their schools, universities, and communities; and initiate research of significance to their professions and the communities served by their institutions.

## Specialty Nursing Organization Resources and Curricula

The National Nurses Society on Addictions (NNSA), the Drug and Alcohol Nurses Association (DANA), and the National Consortium for Chemical Dependency Nurses (NCCDN) promote the roles of nurses in identifying and treating people with alcohol and other drug problems. These organizations conduct continuing education in regional chapters and in the context of annual meetings. NNSA and NCCDN also have developed certifying examinations for nurses specializing in alcohol abuse.

In addition, NNSA published a six-chapter core curriculum of addictions nursing that describes the nursing specialty; reviews etiologic theories; and identifies nursing activities in assessment, intervention, and long-term recovery. The curriculum includes the definition and interpretation of key concepts related to the addictions and their treatment with an emphasis on nursing roles, research innovations, and history of the specialty. Two later volumes published by the NNSA—*National Nurses Society on Addictions Nursing Care Planning with the Addicted Client* (1989)—are more problem oriented. They give specific guidelines on the use of nationally developed standards for nurses working in treatment programs.

An interorganizational publishing effort by the American Nurses Association (ANA), DANA, and NNSA—*Standards of Addictions Nursing Practice with Selected Diagnoses and Criteria* (1988)—outlines an interactive, systematic, problemsolving process that can be used to help patients achieve maximum wellness. Such an approach does not differentiate nursing activities according to the education or skill level of the nurse but rather identifies the patient’s needs and describes steps taken by the nurse to address them. In such a format, the curriculum and the planning guides are useful resources for practicing nurses and continuing education but are less useful in assisting faculty to integrate alcohol abuse knowledge into beginning and advanced courses and programs.

CSAP also has developed a new training system for practicing health professionals, which uses materials from the federally funded curricula and the NNSA core curriculum as part of their onsite, grass-roots training. Courses are designed to assist health professionals in meeting prevention-related needs of parent groups, high-risk youth, community workers, and State agency employees. Through this project, school, community health, and occupational health nurses have access to new information on screening and referral practices.

## Role of Nursing Organizations

Educational resources as well as position statements and standards formulated by the ANA, in collaboration with specialty nursing associations, encourage the development of nursing competence with alcohol-related problems in general practice and in primary care delivery.

The ANA currently is attempting to clarify the role of the advanced nurse practitioner in the delivery of primary care. Nurse clinicians, nurse practitioners, and nurse midwives prepared through certification and master’s degree education use assessment, care delivery, and case management skills, all of which provide avenues for alcohol abuse education and health promotion as well as intervention or referral for abuse and dependence.

Progress is being made toward advancing primary care education about alcohol abuse and related problems in nursing curricula. The keys to success lie in the widespread dissemination of teaching materials that support the comprehensive and sensitive assessment of patients and families who are at risk for alcohol-related problems, especially patients who manifest early or as yet undetected problems.

In addition to developing teaching tools, practitioners, professors, and their associates must have sufficient expertise in the field and be made aware of the need for in-depth evaluation of patients with alcohol or other drug problems in clinical care delivery for progress in alcohol abuse education to continue.

## Footnotes

1 Because the focus of this article is on education about alcohol problems, the terms “alcohol abuse and dependence” and “alcohol-related problems” are used throughout. However, the curricula discussed here also include information on tobacco, drugs, and drug-related problems.

2 A generalist nurse is educated to address health problems and illnesses for patients of all ages and responses during the patient’s life span. These nurses have broad-based experience that is applicable to the entire field of nursing.
